# Global testing of a consensus solubility assessment to enhance robustness of the WHO biopharmaceutical classification system

**DOI:** 10.5599/admet.850

**Published:** 2020-10-07

**Authors:** Valeria Gigante, Giovanni M. Pauletti, Sabine Kopp, Minghze Xu, Isabel Gonzalez-Alvarez, Virginia Merino, Michelle P. McIntosh, Anita Wessels, Beom-Jin Lee, Kênnia Rocha Rezende, Gerhard K.E. Scriba, Gaurav P.S. Jadaun, Marival Bermejo

**Affiliations:** 1 Norms and Standards for Pharmaceuticals, World Health Organization, Geneva, Switzerland; 2 Department of Pharmaceutical and Administrative Sciences, St. Louis College of Pharmacy, St. Louis, Missouri, USA; 3 Institute for Chemical Drug Control, China National Institutes for Food and Drug Control, Beijing, China; 4 Department of Pharmaceutics and Pharmaceutical Technology and Parasitology, University of Valencia, Valencia, Spain; 5 Drug Delivery Disposition and Dynamics, Monash Institute of Pharmaceutical Sciences, Monash University, Parkville, Australia; 6 North_West University, School of Pharmacy, Potchefstroom, South Africa; 7 College of Pharmacy and Institute of Pharmaceutical Science and Technology, Ajou University, Suwon, Republic of Korea; 8 Faculty of Pharmacy, Federal University of Goiás, Brazil; 9 Department of Pharmaceutical Chemistry, Friedrich Schiller-University, Jena, Germany; 10 Department of Engineering: Pharmacy section, Universidad Miguel Hernández de Elche, Alicante, Spain; 11 Indian Pharmacopoeia Commission, Ministry of Health & Family Welfare, Govt. of India, Ghaziabad, India

**Keywords:** biowaiver, multisource products, essential medicines, permeability, regulatory guidance

## Abstract

The WHO Biopharmaceutical Classification System (BCS) is a practical tool to identify active pharmaceutical ingredients (APIs) that scientifically qualify for a waiver of in vivo bioequivalence studies. The focus of this study was to engage a global network of laboratories to experimentally quantify the pH-dependent solubility of the highest therapeutic dose of 16 APIs using a harmonized protocol. Intra-laboratory variability was ≤5 %, and no apparent association of inter-laboratory variability with API solubility was discovered. Final classification “low solubility” vs “high solubility” was consistent among laboratories. In comparison to the literature-based provisional 2006 WHO BCS classification, three compounds were re-classified from “high” to “low-solubility”. To estimate the consequences of these experimental solubility results on BCS classification, dose-adjusted in silico predictions of the fraction absorbed in humans were performed using GastroPlus®. Further expansion of these experimental efforts to qualified APIs from the WHO Essential Medicines List is anticipated to empower regulatory authorities across the globe to issue scientifically-supported guidance regarding the necessity of performing in vivo bioequivalence studies. Ultimately, this will improve access to affordable generic products, which is a critical prerequisite to reach Universal Health Coverage.

## Introduction

In a world where one third of the population lacks access to essential medicines [1] and medicines represent the largest family expenditure after food, generic competition and differential pricing are major drivers to sustain a healthy population, especially in low-income countries [2]. Therefore, the World Health Organization (WHO) has identified access to essential medicines as one of its key priorities to meet the millennium Sustainable Development Goals and reach Universal Health Coverage.

In general, regulatory agencies carry the public health responsibility to carefully balance access to and safety of medicines within their respective authorities. Successful accomplishment of this objective requires risk management strategies that are based on scientifically valid data. The Biopharmaceutical Classification System (BCS) introduced in 1995 [3] recognizes the fundamental contribution of aqueous solubility of an active pharmaceutical ingredient (API) within the physiologically relevant gastrointestinal pH range and its mucosal permeability in governing oral absorption *in vivo*. Consequently, various regulatory agencies around the world have adopted guidances specifying the use of the BCS as a scientific framework to grant a waiver of mandatory human *in vivo* bioequivalence studies for immediate-release solid oral dosage. Hence, qualifications defined for a biowaiver directly impact access to and affordability of a finished pharmaceutical product while assuring the quality of the medicine.

The WHO recognizes the possibility of waiving *in vivo* bioequivalence studies (i.e., biowaiver) as an effective regulatory strategy to accelerate development of generic products for eligible APIs in order to improve access and save lives. Therefore, as part of its 2006 *Guidance on Waiving of Bioequivalence Requirements for Immediate-release Oral Solid Dosage Forms*, the WHO published a provisional classification of APIs based on the 14^th^ WHO Essential Medicines List (EML) with regards to their eligibility for a biowaiver. As this initial list was largely based on literature data, which can vary widely due to different experimental conditions employed, the WHO Expert Committee on Specifications for Pharmaceutical Preparations recommended in 2016 that the WHO revises this list to the current EML [4] using experimentally determined laboratory data according to a globally harmonized protocol in order to facilitate effective generic development, including regulatory review by national regulatory authorities.

The primary objective of the WHO biowaiver guidance is to establish a scientific framework whereby the risk of *bioinequivalence* is reduced to an acceptable level. Leveraging an API’s main physicochemical characteristics that influence intestinal absorption, its clinical safety and efficacy profile, and comparative consideration of the finished pharmaceutical product, it is accepted that an API’s solubility, permeability, and comparative dissolution data of the finished pharmaceutical product represent adequate scientific evidence to enable an informed decision whether or not a biowaiver could be safely recommended for a specific API. Eligible substances are those assigned to Class I (highly soluble and highly permeable) or Class III (highly soluble and low permeable) according to the BSC framework [5].The BCS is widely accepted by various regulatory authority, including EMA and FDA, and highlights the pivotal role of solubility underpinning the biowaiver approach. According to the WHO, an API is considered highly soluble when the highest single therapeutic dose (e.g., the maximum dose administered orally at once) as specified in the approved product label of the originator is soluble in 250 mL or less of aqueous media over the pH range of 1.2–6.8 [6]. Comparative dissolution data are expected to be generated by pharmaceutical manufactures for eligible finished pharmaceutical product containing the APIs classified within this research project. Regulators will assess the similarity in the dissolution profiles generated in order to evaluate a potential waiver from *in vivo* BE studies.

Following a recommendation from the WHO Expert Committee on Specifications for Pharmaceutical Preparations, the WHO Norms and Standards for Pharmaceuticals team initiated in 2017 the WHO Biowaiver Project aimed at revising its 2006 BCS [6]. To support revision of this provisional classification list, which was largely based on secondary aqueous solubility references and a diverse array of permeability data published in the literature, a global multi-center research team was assembled and tasked to study solubility profiles of selected APIs using a harmonized experimental protocol. The project began in 2018 as a small-scale pilot (Cycle I) focusing on the development of a universal, scientifically sound methodology to consistently assess an API’s solubility within the desired gastrointestinal pH range of 1.2–6.8. Preliminary validation of the refined equilibrium solubility protocol was performed by three independent laboratories and details of the procedure were published as the “*WHO Protocol to conduct equilibrium solubility experiments for the purpose of Biopharmaceutics Classification System-based classification of Active Pharmaceutical Ingredients for biowaiver*”, hereinafter referred as the protocol. To prioritize APIs for the second phase of the project (Cycle II), which was intended to compare inter-laboratory variability on API solubility assessment and classification using the globally harmonized protocol, the following selection criteria were defined [7]: i) API included in medicines listed on the EML; ii) intended to be formulated as immediate-release, solid oral dosage forms; iii) relevant to therapeutic areas of major public interest; and iv) exhibiting well-characterized physicochemical properties. Pharmaceuticals of primary interest to the WHO are those associated with WHO Programs focusing on non-communicable diseases, neglected tropical diseases, and maternal-child health, including tuberculosis, malaria, and HIV/AIDS. Candidate APIs for Cycle II of the WHO Biowaiver Project were identified through a prioritization exercise in collaboration with the WHO Prequalification of Medicines Assessment team. Final selection of the API training set for this study was performed after public consultation according to stakeholders’ needs and priorities. Table 1 summarizes the main properties of the selected APIs that were distributed to 10 different international laboratories located in WHO Member States representing the African Region, Region of the Americas, South-East Asian Region, European Region, and Western Pacific Region, respectively.

## Experimental

### Materials

All drug substances used in this study were donated by various suppliers and compliant with compendial specifications as outlined in The International Pharmacopoeia [8] and other national or regional pharmacopoeias. Each API was provided to multiple laboratories according to a risk-based distribution plan that assured experimental solubility assessment by three independent facilities. Controlled substances, which are difficult to ship across borders, were measured in two facilities. Table 1 summarizes API-specific information regarding therapeutic indication, selected physicochemical properties, and the provisional BCS classification assigned by the WHO in 2006 based on solubility and permeability data obtained from the primary literature. All chemicals selected for experimental solubility assessments were of high purity or analytical grade and were used as received.

### Solubility assessment

Solubility of the highest therapeutic dose according to the approved label or summary of product characteristics of the originator was determined for each API at 37 ± 1 °C in aqueous buffer solutions at pH 1.2, 4.5, and 6.8 using a globally harmonized protocol [9]. Across the various laboratories engaged in this study, equilibrium solubility was quantified by the “shake flask” method supported by a validated, stability-indicating analytical methodology such as high-performance liquid chromatography.

The time required to reach equilibrium was experimentally defined during a preliminary experiment performed at the pH value where lowest API solubility was predicted. Subsequently, pivotal experiments were initiated in triplicate at pH 1.2, 4.5, and 6.8, respectively, using approximately a 10-50 % excess amount of the API estimated to meet the WHO “high solubility” class boundary (see below). Undissolved solid was separated by either filtration or centrifugation prior to API quantitation employing a validated, stability-indicating analytical methodology. Individual solubility was reported in mg/mL. As specified in the WHO Technical Report Series No. 1003 Annex 7 entitled “Multisource (generic) pharmaceutical products: guidelines on registration requirements to establish interchangeability” [5], an API is considered “highly soluble” when the dose/solubility volume (DSV) representing the volume of liquid necessary to completely dissolve the highest single therapeutic dose of the API as recommended by the approved label or summary of product characteristics of the originator product is consistently smaller than 250 mL over the entire pH-range of 1.2 – 6.8.

### Permeability data

To estimate the consequences of the experimental solubility results generated by this global laboratory consortium using the harmonized WHO protocol on BCS classification of each API, it was necessary to obtain API-specific permeability data indicative of the fraction absorbed in humans (*f*_a_). To limit variability and selection bias associated with different experimental conditions used to define API permeability as shown by Larregieu and Benet for Caco-2 cell permeability assessment [10], a consistent computational simulation approach was implemented using GastroPlus®9.7 (Simulations Plus, Inc., Lancaster, CA) under a free academic license. GastroPlus®9.7 is a bottom-up, whole-body physiologically-based pharmacokinetic (PBPK) model that uses the Advanced Compartmental Absorption Transit (ACAT) mechanistic absorption model to mimic the human intestinal absorption of oral formulations from the GI tract [11]. Simulations were conducted for the highest therapeutic dose as well as the highest strength of each API reported in Table 1 using the human fasted physiological model for an individual with an average bodyweight of 70 kg without enterohepatic recirculation following administration of an immediate release tablet associated with a default gastric transit time of 15 min. Gastrointestinal absorption rate in the ACAT model is estimated over a physiological pH range of 1.3 – 6.8 employing an absorption scale factor logD model, which considers regional permeability according to predicted physiological changes in trans- and paracellular transport, anatomical changes in surface area due to the presence of villi and microvilli, and gastrointestinal pH gradient. A summary of general input parameters used for this PBPK modeling approach is provided in Table 2. API-specific input parameters were generated in silico using the ADMET Predictor^©^v9.5.0.0 (Simulations Plus, Inc., Lancaster, CA). To compare *in silico* predicted *f*_a_ values for gastrointestinal absorption in humans to clinically determined oral bioavailability data indicative of the fraction absorbed *in vivo*, a literature search was performed in the PubMed database (www.ncbi.nlm.nih.gov) using the international nonproprietary name of the selected API in combination with one or more of the following search terms: absorption, BCS, bioavailability, fraction absorbed, gastrointestinal, isotope, mass balance, oral, and pharmacokinetics. Moreover, publicly available documents associated with regulatory approvals of originator and generic drug products for the respective API were consulted to collect pertinent *in vivo* pharmacokinetic information from human trials.

### Statistical analysis

All experiments were carried out at least in three independent laboratories, and results are reported as mean ± standard deviation (SD). Coefficients of variation (%CV) were calculated as 100×SD/mean. Statistically significant differences (p<0.05) between groups were evaluated using unpaired Student’s *t*-test or one-way analysis of variance (ANOVA) where appropriate (Microsoft Excel 2010, Microsoft, Redmond, WA).

## Results and discussion

### Complexities of solubility measurements

A distinct feature of the WHO BCS when compared to other BCS classification systems as defined by various regional regulatory authorities is that the WHO classification centers on the highest single oral therapeutic dose of each APIs (as recommended by the approved label/summary of product characteristics of the originator) to estimate the DSV. The rationale for this definition is that BCS classification is mimicking more closely real life conditions and corresponds to the “worst case scenario” when patients are taking the therapeutic oral dose prescribed. Moreover, a focus on the highest single oral dose provides the necessary flexibility to consider different strengths available on the market when regulatory decisions regarding a biowaiver are discussed. Overall, these measures are consistent with the WHO’s priority on facilitating access to affordable medicines globally.

In general, samples are expected to reach equilibrium within 24 hours. However, the time required to arrive at equilibrium between solid and dissolved API may be influenced by various experimental factors ranging from intrinsic API characteristics, excess of solid included, to the type and speed of agitation method used [12]. This may leads to long dissolution times until saturation is reached that carry the risk of introducing structural changes in the drug substance when in contact with buffers solutions, including transition into a different polymorphic state, salt or solvate. In solution, salt forms are more likely to re-crystallize resulting in highly stable molecular configurations (i.e., free forms) with significantly altered dissolution properties. For ionized molecules, in addition, the propensity for self-aggregation and/or complexation is increased while in contact with buffer components.

The conventional shake-flask method represents the “gold standard” for solubility assessment as it is simple and easy to perform and only requires inexpensive instrumentation. When executed according to a well-designed protocol, it generally results in high quality data, with standard deviation ≤5 %. However, the shake-flask method is also time- and labor-intensive and may consume significant amounts of drug substance in order to measure thermodynamic solubility of a highly soluble API in the presence of its solid form. To minimize such constraints, the harmonized protocol defines the highest API concentration for pivotal solubility determination in this study to a concentration representing at least twice the highest therapeutic dose over 250 mL of buffer. Consequently, the experimentally determined concentration value does not represent a true “equilibrium” solubility but rather assures that DSV<1, which is compliant with the definition of “high solubility”.

The results of this global solubility assessment for each API are summarized in Table 3. It is important to re-emphasize that individual laboratories did not attempt to quantify the thermodynamic equilibrium solubility (i.e., saturation concentration in the presence of excess solid). Therefore, for APIs with high solubility, recorded values were measured after complete dissolution of the solid. Consequently, greater variability in absolute API concentrations recorded in Table 3 under specified pH conditions is the result of different API amounts used for the solubility experiments rather than a demonstration of highly variable techniques implemented by the different laboratories.

Among the 10 APIs that were previously included in the 2006 WHO BCS, which was largely based on secondary aqueous solubility references, experimental values reported from three independent laboratories unambiguously justified re-classification of aciclovir, amoxicillin, and ethionamide from a “high solubility” (HS) to a “low solubility” (LS) drug. The change in classification of amoxicillin and aciclovir was related to the solubility assessment performed at the highest therapeutic dose (i.e., 3,000 mg for amoxicillin and 800 mg for aciclovir) instead of the highest strength as both drugs exhibit dose-dependent solubility profiles [13,14].

### Inter-laboratory reproducibility

**Figure 1.**
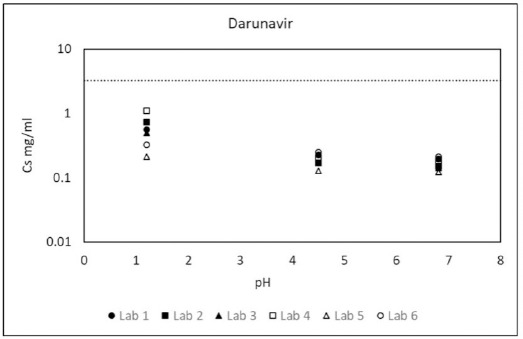
Experimental pH-dependent solubility values reported for darunavir by six different laboratories. Dotted line represents the class boundary between “high solubility” and “low solubility” for the highest therapeutic dose of darunavir (i.e., 800 mg)

To assess the robustness of the globally harmonized solubility protocol, each API was analyzed by at least three different laboratories. The only exception was codeine, which was analyzed by two different laboratories due to its controlled substance status that limited distribution across borders. Figure 1 illustrates the variability in pH-dependent solubility data reported for darunavir, as a representative example, which was analyzed by six different laboratories. Laboratory-specific interpretation of experimental parameters that were deliberately kept flexible in the globally harmonized protocol to enable wide spread adoption across various resource settings [15] introduces unavoidable variability in absolute solubility data, ranging between 20-50 %. However, it is important to note that intra-laboratory variability was always ≤5 % and the final solubility classification of darunavir as a “low solubility” drug was consistent among all laboratories. Similar consistencies were observed for all other APIs (see Supplemental Information).

**Figure 2.**
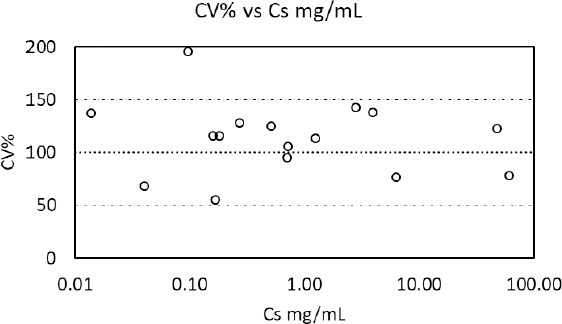
Observed inter-laboratory variability versus average solubility values across experiments

Avdeef and co-workers examined over 800 publications describing equilibrium solubility assessment of sparingly soluble ionizable drug-like molecules by the shake-flask and related methods [16]. This comprehensive analysis identified many factors that affect the quality of the experimental outcome. Among all studies, the reported standard deviation among laboratories varied from 0.17 to 0.58 [17] log units with reported values up to 1.48 log units for some sparingly soluble compounds. It is predicted that a standardized protocol can control some of this inter-laboratory variability. However, compound-specific factors such as formation of polymorphs, hydrates, solvates, amorphous solids, and the impact of stereoisomers remain sources for experimental variability despite the best intention for consistent experimental execution of a protocol. In contrast, the overall standard deviation among all reported solubility values from the different laboratories in this study was 0.83 log units, but 50 % of the API data fell within a narrower range of 0.7 log units, which is consistent with previous literature comparisons. Figure 2 attempts to visualize this inter-laboratory variability by representing the distribution of solubility data as coefficient of variation (CV%) in comparison to the average observe value (i.e., 100 %). The absence of a defined association between inter-laboratory variability and respective API solubility supports the robustness of the globally harmonized protocol. Most importantly, the overall solubility classification based on individual solubility data was always consistent among all laboratories indicating that key determinants of the experimental approach in this globally harmonized protocol were adequately controlled to allow consistent solubility assessment for the purpose of BCS classification.

**Figure 3.**
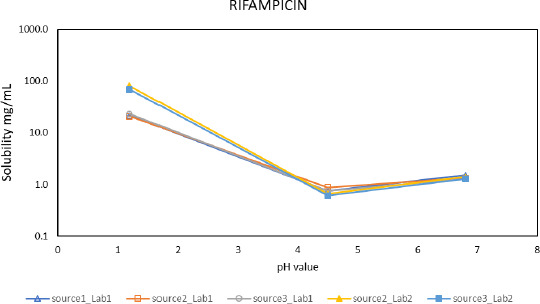
Comparative pH-dependent solubility profile of rifampicin obtained from three different suppliers and analyzed by two different laboratories

To explore the dependence of these solubility assessments on the API source material used for the experimental approach, various laboratories received the same API that was provided by two or three different suppliers. Figure 3 summarizes the results for rifampicin, which was obtained from three different manufacturers and analyzed by two different laboratories. The consistent solubility data obtained for all three batches suggest that the physical properties of the API provided by the different suppliers were highly similar. This conclusion is consistent with the requirement of this study that all APIs must comply with compendial specifications that include physical properties. It is noted that absolute solubility values obtained for the different batches of rifampicin at pH 1.2 exhibit greater variability than measured at pH 4.5 and pH 6.8, respectively. The reason for the higher variability observed at pH 1.2 was probably due to chemical instability that was reported by most laboratories analyzing this API.

### Intestinal Permeability

Harmonization across established biowaiver guidance documents consistently defines the permeability class boundary for “high permeability” as the fraction absorbed (*f*_a_) ≥85 %. However, different regulatory authorities identify either the highest therapeutic dose or the highest marketed formulation strength as the relevant dose that should be considered for the purpose of a biowaiver pathway [18]. To explore the sensitivity of the BCS classification with respect to the permeability of the oral dose administered, computational simulations using GastroPlus® were performed estimating *f*_a_ for the highest therapeutic dose and highest formulation strength of each API.

**Figure 4.**
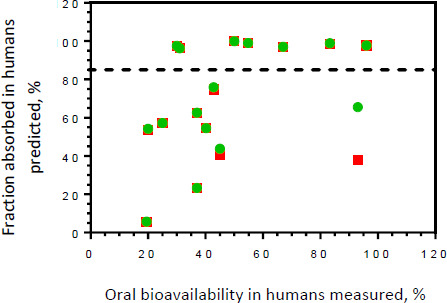
Intestinal Permeability Classification. Fraction absorbed in humans after oral administration of an immediate-release tablet was predicted for the highest daily dose (red squares) and the highest formulation strength (green circles) using GastroPlus®. These simulated predictions were compared with results from clinical oral bioavailability studies performed in humans (see Table 3 for references). The “high permeability” class boundary at the fraction absorbed (*f*a) = 85 % is represents by the dashed line

The results from this *in silico* modeling approach that utilized a consistent set of model input variables as outlined in Table 2 are summarized in Figure 4. The almost perfect overlay between the two symbols representing the different dose levels for most of the selected APIs in this training set suggests that biopharmaceutical properties remain quite constant, irrespectively whether the highest therapeutic dose or the highest formulation strength is modeled. In the context of the BCS, this result implies that gastrointestinal solubility and permeability remain unchanged within the two different dose levels compared. Mechanistically, this dose proportionality also supports the hypothesis that gastrointestinal absorption for most of these APIs is predominantly driven by passive diffusion. Interestingly, the visual representation of these computational simulations identifies two notable exceptions. The predictions for amoxicillin estimate a *f*_a_ = 43.9 % for the 500 mg strength, whereas the predicted *f*_a_ for a therapeutic oral dose of 3,000 mg is only 40.4 %. Although this small reduction in the predicted *f*_a_ for 3,000 mg dose may need confirmation using a broader set of simulation parameters, it seems consistent with results from human pharmacokinetic studies that identified nonlinear absorption kinetics for this penicillin-type antibiotic after oral administration [19]. Mechanistic evaluation of intestinal permeation pathways contributing to oral absorption of amoxicillin revealed a saturable, capacity-limited component mediated by the intestinal oligopeptide transporter, PEPT1 [20]. The second API in this training set predicted to exhibit dose-dependent absorption kinetics is rifampicin. For the 300 mg strength, the estimated *f*_a_ = 65.6 % but dramatically decreases to *f*_a_= 37.9 for a therapeutic oral dose of 750 mg. Mariappan and Singh assessed the mechanisms underlying variable gastrointestinal absorption of rifampicin using a rat model [21]. The results from this preclinical study provided evidence for limited transfer of this antituberculosis agent across the mucosal barrier in a regio-specific manner (jejunum > ileum) due to significant affinity for intestinal efflux systems such as P-glycoprotein. Moreover, it was discovered that transepithelial flux of rifampicin in the duodenum increased up to limit of 300 μg/mL and becoming constant thereafter, which is indicative of a saturable absorption component such as an influx transporter. Since the predicted f_a_ for either the highest therapeutic dose or highest formulation strength of amoxicillin and rifampicin were <85 %, both APIs were qualified as “low permeability” drugs when using the computationally predicted permeability parameter for the purpose of BCS classification.

The results from these exploratory *in silico* permeability predictions using default simulation algorithms of GastroPlus^©^ identified seven APIs from this training set, namely codeine, daclatasvir, dolutegravir, ethionamide, primaquine, pyrimethamine, and raltegravir as “high permeability” drugs according to the WHO BCS threshold of *f*_a_ ≥85 %. In comparison to clinically determined human bioavailability (*F*) data for these “high permeability” drugs identified from the primary literature, the variability in the fractions of the drug reaching the systemic circulation in vivo (30.0 % ≤ *F* ≤ 96.0 %) is significantly greater than the computationally predicted fraction absorbed using GastroPlus^©^ (95.8 % ≤ *f*_a_ ≤ 99.8 %). This is an important reminder of the critical role of model input data that are used to perform PBPK simulations. GastroPlus© was used in this study as an example to illustrate the potential of a bottom-up PBPK simulation tool when exploring BCS classification of APIs for which no clinical bioavailability data are available. Nevertheless, default simulation parameters as outlined in Table 2 may not adequately represent the specific conditions encountered in vivo. This may include important volumetric terms of gastrointestinal segments that can affect drug solubility and/or precipitation rate of a free salt form and a solvate. Alternatively, physiological contribution of in- and efflux transporters that have the ability to significantly modulate the extent and rate at which a drug substance becomes available in the systemic circulation after oral administration (= bioavailability) may not be adequately represented by default model parameters. Similarly, gene polymorphism responsible for different expression levels of major metabolism enzymes (see Supplemental Information) that can lead to variable pre-systemic elimination of the API at the brush-border membrane may not be appropriately considered when using default model parameters. Therefore, various research groups have already initiated comprehensive research studies to identify critical model input parameters that must be defined in order to enhance the in vivo relationship and bioequivalence prediction using PBPK absorption models [22,23].

### API Assignment to WHO Biopharmaceutical Classification System

The main objective of this study was to validate provisional API classification performed earlier using literature-based data (i.e., 2006 WHO BCS) with an updated experimental solubility assessment that was based on a globally harmonized protocol. Table 4 summarizes the results from this study and compares provisional API assignments to the WHO BCS.

Completion of equilibrium solubility experiments by a consortium of 10 international laboratories located in different WHO Regions provided the scientific basis to change the solubility designation from “high solubility” to “low solubility” for aciclovir, amoxicillin, and ethionamide. This result may have been the consequence of updated highest therapeutic dose values when compared to the provisional 2006 WHO BCS classification. In addition, the results from this study provided qualified solubility data for five APIs (i.e., daclatasvir, darunavir, dolutegravir, raltegravir, and tenofovir disoproxil fumarate) that were not considered during the previous WHO BCS assignment in 2006. For the remaining six APIs, experimental solubility assessment confirmed the earlier solubility classification based on literature data. However, due the experimental difficulty to directly assess *f*_a_ in humans, the research community is faced with widespread uncertainty regarding the most appropriate methodology to consistently categorize APIs as “high permeability” or “low permeability” drugs [39]. Provisional classification of APIs according to the WHO BCS in 2006 [6] was exclusively based on literature data. Ambiguity regarding the permeability classification was noted in 28.2 % of all 131 APIs examined.

To explore alternative options that may overcome limitations of literature-based permeability classification, various research groups have reported the use of computational PBPK absorption models for bioequivalence predictions [22,23]. In this study, the GastroPlus^©^ platform was used as an example to illustrate the potential of a bottom-up PBPK simulation tool when exploring BCS classification of APIs, particularly in situations when clinical bioavailability data are not available. The results from this *in silico* exercise are consistent with previous literature-based permeability assignments for aciclovir, furosemide, and primaquine. Furthermore, adoption of this *a priori* modeling approach using default GastroPlus^©^ input parameters (see Table 2), combined with clinical bioavailability data generated for different oral doses, made it feasible to define with high confidence permeability for darunavir and tenofovir disoproxil, which were not considered during the previous WHO BCS assignment in 2006. Similarly, using the same consensus approach that compares the in silico predicted *f*_a_ in humans and clinically determined bioavailability data for the same API but measured at different doses, the ambiguous BCS classification of azithromycin, efavirenz, and furosemide were clarified. However, the results summarized in Table 4 clearly underline significant discrepancies between PBPK-predicted human absorption data and clinically measured bioavailability values, specifically for codeine, dolutegravir, ethionamide, pyrimethamine, raltegravir, and rifampicin. For some of those APIs, such as rifampicin, which has been described to experience regio-specific absorption (jejunum > ileum) due to significant affinity for intestinal efflux systems such as P-glycoprotein [21], the literature may provide clues for a reasonable scientific hypothesis that could explain these inconsistent results. For other APIs, scientific explanations are less clear and may require further exploration using carefully designed experimental approaches to assess permeability. It is generally assumed that physiologically-based *in silico* absorption models are promising supplemental tools to traditional *in vitro* assays and preclinical *in vivo* studies. However, as recently reported by Sjögren and colleagues [40], different modeling platforms are associated with inconsistent over- and under-prediction of the fraction absorbed. Consequently, a broader data mining approach will be necessary to maximize confidence in model performance.

## Conclusions

The results from this study demonstrate successful validation of a globally harmonized solubility protocol across different international laboratories to support WHO BCS classification. Implementation of a consensus approach that compares in silico predicted *f*_a_ in humans using the GastroPlus^©^ PBPK simulation platform and clinically determined bioavailability data for the same API but measured at different doses enabled unambiguous assignment of 10 out of the 16 APIs selected for this pilot study to one of the four BCS classes. Further expansion of these experimental efforts to qualified APIs from the WHO Essential Medicines List is predicted to provide regulatory authorities across the globe with scientifically validated data to support decisions regarding the need for *in vivo* bioequivalence studies. Ultimately, this will improve access to affordable generic products, which is a critical prerequisite to reach Universal Health Coverage.



## Figures and Tables

**Table 1.** Active pharmaceutical ingredients prioritized for WHO BCS classification in Cycle II

API (solid form listed in EML)	Therapeutic area	Indication^a^	Highest Therapeutic Dose [mg]^b^	Highest Strength [mg]	2006 WHO Provisional BCS ^c^	log*P*^d^	*M*_W_^e^ [g/mol]	Number of suppliers
Aciclovir	Antiviral medicines	Antiherpes medicines	800	200	III	-1.40	225.2	1
Amoxicillin (trihydrate)	Antibacterials	Antibiotics	3000	500	I	-1.94	419.5	2
Azithromycin (dihydrate)	Antibacterials	Antibiotics	2000	500	IV/II	3.39	785.0	1
Cefixime (trihydrate)	Antibacterials	Antibiotics	400	400	IV	-1.23	507.5	1
Codeine (phosphate hemihydrate) (2:2:1)	Medicines for pain and palliative care	Opioid analgesics	60	30	III	1.61	812.7	1
Daclatasvir (dihydrochloride)	Antiviral medicines	Medicines for hepatitis C	60	60	Not classified	4.06	811.8	2
Darunavir (ethanolate)	Antiviral medicines	Antiretrovirals (HIV)	800	800	Not classified	1.93	593.7	2
Dolutegravir	Antiviral medicines	Antiretrovirals (HIV)	50	50	Not classified	1.20	419.4	1
Efavirenz	Antiviral medicines	Antiretrovirals (HIV)	600	600	IV/II	4.19	315.7	3
Ethionamide	Antibacterials	Antituberculosis medicines	500–1000	250	III/I	1.26	166.3	1
Furosemide	Cardiovascular medicines	Medicines used in heart failure	80	40	IV/II	2.07	330.7	2
Primaquine (phosphate) (1:2)	Antiprotozoal medicines	Antimalarial medicines (curative treatment of *P.vivax* and *P.ovale* infections)	15	15	I	2.86	455.3	2
Pyrimethamine	Antiprotozoal medicines	Antimalarial medicines	75	25	IV/III	2.53	248.7	3
Raltegravir (potassium)	Antiviral medicines	Antiretrovirals (HIV in pregnant women and in second-line)	400	400	Not classified	1.97	482.5	2
Rifampicin	Antibacterials	Antituberculosis/Antileprosy medicines	750	300	II	2.53	822.9	3
Tenofovir disoproxil fumarate (1:1)	Antiviral medicines	Antiretrovirals (HIV)	300	300	Not classified	-1.34	635.5	1

^a^ According to the 21^st^*WHO Model List of Essential Medicines* (2019)

^b^ According to Summary of Product Characteristics from WHO-PQ or National/Regional Regulatory Authority

^c^ Proposal to waive in vivo bioequivalence requirements for WHO Model List of Essential Medicines immediate-release, solid oral dosage forms. In: WHO Expert Committee on Specifications for Pharmaceutical Preparations: fortieth report. Geneva: World Health Organization; 2006: Annex 8 (WHO Technical Report Series, No. 937; https://www.who.int/medicines/areas/quality_safety/quality_assurance/ProposalWaiveVivoBioequivalenceRequirementsModelListEssentialMedicinesImmediateReleaseSolidOralDosageFormsTRS937Annex8.pdf?ua=1, accessed 4 May 2020). *Updated requirement in:* Multisource (generic) pharmaceutical products: guidelines on registration requirements to establish interchangeability. In: WHO Expert Committee on Specifications for Pharmaceutical Preparations: fifty-first report. Geneva: World Health Organization; 2017: Annex 6 (WHO Technical Report Series, No. 1003; https://www.who.int/medicines/areas/quality_safety/quality_assurance/trs1003_annex6.pdf?ua=1, accessed 2 October 2020).

^d^ In silico log *P* values predicted using ADMET Predictor® 9.5 (Simulations Plus, Inc., Lancaster, CA)

^e^ The molecular weight corresponds to the solid form of the API used for the solubility experiments as indicated in column 1 of this table.

**Table 2.** General Formulation and PBPK Simulation Parameters used as Model Input Variables in GastroPlus®

Input Parameter	Value
Dosage Form	Immediate release tablet
Dose	Highest therapeutic dose/formulation strength
Dose volume [mL]	250
Mean precipitation time [s]	900
Drug particle density [g/mL]	1.2
Mean particle radius [μm]	25
Number of bins	1
Shape factor	1
Dissolution model	Johnson
Bile salt effect	Off
PBPK model	Human fasted (male, 30 years, 70 kg)
Small intestine transit time [h]	3.2
ASF model	OptlogD Model SA/V 6.1
Paracellular model	Zhimin
Biliary clearance fraction	0
Simulation mode	single
Simulation time [h]	24

**Table 3.** Experimentally determined pH-dependent API solubility using a globally harmonized protocol

API	pH	Cs mean mg/mL^a^	SD	CV%	DSV	Solubility class
Aciclovir	1.2	4.14	4.71	113.87	193.44	LS
	4.5	1.25	1.11	88.96	642.57	
	6.8	1.27	1.10	86.45	628.93	
Amoxicillin (trihydrate)	1.2	9.68	13.84	142.96	206.64	LS
	4.5	2.78	1.64	58.96	718.17	
	6.8	2.84	2.51	88.38	703.69	
Azithromycin (dihydrate)	1.2	9.51	NA	NA	210.39	LS
	4.5	6.32	4.83	76.42	316.53	
	6.8	6.43	0.08	1.25	310.90	
Cefixime (trihydrate)	1.2	0.72	0.76	106.07	555.56	LS
	4.5	4.44	4.31	97.12	90.15	
	6.8	7.45	4.43	59.46	53.66	
Codeine (phosphate hemihydrate)	1.2	60.68	42.18	69.52	0.99	HS
	4.5	66.86	51.12	76.46	0.90	
	6.8	70.38	54.91	78.02	0.85	
Daclatasvir (dihydrochloride)	1.2	88.17	125.07	141.86	0.68	LS
	4.5	1.78	3.48	195.74	33.72	
	6.8	0.10	0.14	142.09	624.09	
Darunavir (ethanolate)	1.2	0.57	0.32	55.56	1402.40	LS
	4.5	0.19	0.04	23.30	4202.05	
	6.8	0.17	0.03	20.14	4776.12	
Dolutegravir	1.2	0.04	0.03	68.10	1252.09	LS
	4.5	0.04	0.02	61.99	1273.34	
	6.8	0.05	0.02	44.62	943.99	
Efavirenz	1.2	0.18	0.20	109.87	3331.65	LS
	4.5	0.20	0.24	115.60	2947.17	
	6.8	0.21	0.24	114.51	2857.39	
Ethionamide	1.2	10.23	12.82	125.24	24.43	LS
	4.5	0.96	0.16	16.71	259.97	
	6.8	0.51	0.08	15.29	488.28	
Furosemide	1.2	0.01	0.02	137.59	5818.26	LS
	4.5	0.14	0.05	36.66	558.95	
	6.8	3.74	2.04	54.39	21.37	
Primaquine (phosphate)	1.2	50.51	62.22	123.19	0.30	HS
	4.5	51.38	63.55	123.67	0.29	
	6.8	48.16	59.06	122.63	0.31	
Pyrimethamine	1.2	1.70	0.85	50.17	44.24	LS
	4.5	4.91	3.98	80.98	15.27	
	6.8	0.27	0.35	128.09	277.50	
Raltegravir (potassium)	1.2	0.16	0.18	110.85	2513.14	LS
	4.5	0.17	0.15	91.02	2363.23	
	6.8	0.48	0.56	115.85	826.45	
Rifampicin	1.2	41.36	39.37	95.18	14.51	LS
	4.5	0.70	0.39	55.54	851.79	
	6.8	1.15	0.61	52.93	521.92	
Tenofovirdisoproxil fumarate	1.2	9.24	12.79	138.44	32.48	HS
	4.5	3.94	3.46	87.78	76.14	
	6.8	4.35	4.53	104.12	68.97	

*^a^ Experimental data represent mean values of three individual experiments*

**Table 4.** Comparison of provisional API assignment to WHO BCS

API	Highest Therapeutic Dose [mg]^a^	Provisional 2006 WHO BCS^b^	Experimentally Assigned Solubility Class	GastroPlus^©^-predicted Fraction Absorbed in Humans	Oral Bioavailability in Humans [%]^c^	Provisional 2020 WHO BCS
Aciclovir	800	III	LS	LP	*20.0 [24]	IV
Amoxicillin trihydrate	3000	I	LS	LP	45.0 [19]	IV
Azithromycin dihydrate	2000	IV/II	LS	LP	*37.0 [25]	IV
Cefixime trihydrate	400	IV	LS	LP	40.2 [26]	IV
Codeine phosphate semihydrate	60	III	HS	HP	54.8 [27]	I/III
Daclatasvir dihydrochloride	60	Not classified	LS	HP	67.0 [28]	II/IV
Darunavir ethanolate	800	Not classified	LS	LP	*37.0 [29]	IV
Dolutegravir	50	Not classified	LS	HP	31.0 [30]	II/IV
Efavirenz	600	II/IV	LS	LP	19.5 [31]	IV
Ethionamide	500–1000	III/I	LS	HP	*83.3 [32]	II/IV
Furosemide	80	IV/II	LS	LP	*42.8 [33]	IV
Primaquine phosphate	15	I	HS	HP	96.0 [34]	I
Pyrimethamine	75	IV/III	LS	HP	*50.0 [35]	II/IV
Raltegravir potassium	400	Not classified	LS	HP	30.0 [36]	II/IV
Rifampicin	750	II	LS	LP	*93 [37]	II/IV
Tenofovir disoproxil fumarate	300	Not classified	HS	LP	25 [38]	III

^a^According to Summary of Product Characteristics from WHO-PQ or National/Regional Regulatory Authority.

^b^ Proposal to waive in vivo bioequivalence requirements for WHO Model List of Essential Medicines immediate-release, solid oral dosage forms. In: WHO Expert Committee on Specifications for Pharmaceutical Preparations: fortieth report. Geneva: World Health Organization; 2006: Annex 8 (WHO Technical Report Series, No. 937; https://www.who.int/medicines/areas/quality_safety/quality_assurance/ProposalWaiveVivoBioequivalenceRequirementsModelListEssentialMedicinesImmediateReleaseSolidOralDosageFormsTRS937Annex8.pdf?ua=1, accessed 4 May 2020). *Updated requirement in:* Multisource (generic) pharmaceutical products: guidelines on registration requirements to establish interchangeability. In: WHO Expert Committee on Specifications for Pharmaceutical Preparations: fifty-first report. Geneva: World Health Organization; 2017: Annex 6 (WHO Technical Report Series, No. 1003; https://www.who.int/medicines/areas/quality_safety/quality_assurance/trs1003_annex6.pdf?ua=1, accessed 2 Oct 2020).

^C^ oral bioavailability data were obtained from the primary literature. LS = low solubility; HS = high solubility; LP = low permeability; HP = high permeability

* clinical bioavailability data using an oral dose different than the highest therapeutic dose listed in Table 1

## References

[1] World Health Organization, Access to medicines: making market forces serve the poor. Ten years in public health 2007–2017., (2017). https://www.who.int/publications/10-year-review/en/ (accessed May 6, 2020).

[2] The World Medicines Situation: Chapter 7. Access to essential medicines, (1999). http://digicollection.org/hss/en/d/Js6160e/9.html (accessed May 6, 2020).

[3] Amidon G.L. Lennernäs H. Shah V.P. Crison J.R., A Theoretical Basis for a Biopharmaceutic Drug Classification: The Correlation of in Vitro Drug Product Dissolution and in Vivo Bioavailability, Pharm. Res. An Off. J. Am. Assoc. Pharm. Sci. 12 (1995) 413–420. doi: https://doi.org/10.1023/A:1016212804288. 10.1023/A:1016212804288 7617530

[4] World Health Organization, The Selection and Use of Essential Medicines The Selection and Use of Essential Medicines WHO Technical Report Series, Geneva, 2019. http://www.who.int/ (accessed May 6, 2020).

[5] World Health Organization, Multisource (generic) pharmaceutical products: guidelines on registration requirements to establish interchangeability, in: WHO Expert Comm. Specif. Pharm. Prep., Geneva, 2017. https://www.who.int/medicines/areas/quality_safety/quality_assurance/expert_committee/WHO_TRS_1003_full-version.pdf?ua=1 (accessed May 6, 2020).

[6] World Health Organization, Proposal to waive in vivo bioequivalence requirements for WHO Model List of Essential Medicines immediate-release, solid oral dosage forms, 2006. https://www.who.int/medicines/areas/quality_safety/quality_assurance/ProposalWaiveVivoBioequivalenceRequirementsModelListEssentialMedicinesImmediateReleaseSolidOralDosageFormsTRS937Annex8.pdf?ua=1 (accessed May 6, 2020).

[7] World Health Organization, WHO Expert Committee on Specifications for Pharmaceutical Preparations The Expert Committee on Specifications for Pharmaceutical, World Health Organization, Geneva, 2020. https://apps.who.int/iris/handle/10665/331814 (accessed May 6, 2020).

[8] World Health Organization, The International Pharmacopoeia, Ninth Edition, (2019). https://apps.who.int/phint/en/p/docf/ (accessed May 6, 2020).

[9] World Health Organization, Annex 4 Protocol to conduct equilibrium solubility experiments for the purpose of Biopharmaceutics Classification System-based classification of active pharmaceutical ingredients for biowaiver, in: WHO Expert Comm. Specif. Pharm. Prep. Fifty-Third Rep., 2019. https://www.who.int/medicines/areas/quality_safety/quality_assurance/WHO_TRS_1019_Annex4.pdf?ua=1 (accessed May 6, 2020).

[10] Larregieu C.A. Benet L.Z. Drug discovery and regulatory considerations for improving in silico and in vitro predictions that use caco-2 as a surrogate for human intestinal permeability measurements. AAPS J. 15 (2013) 483–497. doi: https://doi.org/10.1208/s12248-013-9456-8. 10.1208/s12248-013-9456-8 23344793 PMC3675726

[11] Agoram B. Woltosz W.S. Bolger M.B.. Predicting the impact of physiological and biochemical processes on oral drug bioavailability. Adv. Drug Deliv. Rev. 50 (2001). doi: https://doi.org/10.1016/S0169-409X(01)00179-X. 10.1016/S0169-409X(01)00179-X 11576695

[12] Avdeef A. Fuguet E. Llinàs A. Ràfols C. Bosch E. Völgyi G. Verbic T. Boldyreva E. Takács-Novák K.. Equilibrium solubility measurement of ionizable drugs - consensus recommendations for improving data quality. ADMET DMPK 4 (2016) 117–178. doi: https://doi.org/10.5599/admet.4.2.292. 10.5599/admet.4.2.292

[13] Thambavita D. Galappatthy P. Mannapperuma U. Jayakody L. Cristofoletti R. Abrahamsson B. Groot D.W. Langguth P. Mehta M. Parr A. Polli J.E. Shah V.P. Dressman J.. Biowaiver Monograph for Immediate-Release Solid Oral Dosage Forms: Amoxicillin Trihydrate. J. Pharm. Sci. 106 (2017) 2930–2945. doi: https://doi.org/10.1016/j.xphs.2017.04.068. 10.1016/j.xphs.2017.04.068 28483422

[14] Arnal J. Gonzalez-Alvarez I. Bermejo M. Amidon G.L. Junginger H.E. Kopp S. Midha K.K. Shah V.P. Stavchansky S. Dressman J.B. Barends D.M.. Biowaiver monographs for immediate release solid oral dosage forms: Aciclovir. J. Pharm. Sci. 97 (2008) 5061–5073. doi: https://doi.org/10.1002/jps.21392. 10.1002/jps.21392 18425814

[15] Ono A. Matsumura N. Kimoto T. Akiyama Y. Funaki S. Tamura N. Hayashi S. Kojima Y. Fushimi M. Sudaki H. Aihara R. Haruna Y. Jiko M. Iwasaki M. Fujita T. Sugano K.. Harmonizing solubility measurement to lower inter-laboratory variance - Progress of consortium of biopharmaceutical tools (CoBiTo) in Japan. ADMET DMPK 7 (2019) 183–195. doi: https://doi.org/10.5599/admet.704. 10.5599/admet.704 PMC8957233 35350659

[16] Avdeef A.. Suggested improvements for measurement of equilibrium solubility-pH of ionizable drugs. ADMET DMPK 3 (2015) 84–109. doi: https://doi.org/10.5599/admet.3.2.193. 10.5599/admet.3.2.193

[17] Katritzky A. Wang Y. Sild S. Tamm T. Karelson M.. QSPR Studies on Pressure Vapor Solubility Aqueous, and the Prediction of Water–Air Partition Coefficients. J. Chem. Inf. Comput. Sci. 38 (1998) 720–725. doi: https://doi.org/10.1021/ci980022t. 10.1021/ci980022t

[18] Hofsäss M.A. Dressman J.B.. The Discriminatory Power of the BCS-Based Biowaiver: A Retrospective With Focus on Essential Medicines. J. Pharm. Sci. 108 (2019) 2824–2837. doi: https://doi.org/10.1016/j.xphs.2019.04.030. 10.1016/j.xphs.2019.04.030 31059698

[19] Paintaud G. Alván G. Dahl M.L. Grahnén A. Sjövall J. Svensson J.O.. Nonlinearity of amoxicillin absorption kinetics in human. Eur. J. Clin. Pharmacol. 43 (1992) 283–288. doi: https://doi.org/10.1007/BF02333024. 10.1007/BF02333024 1425893

[20] Luckner P. Brandsch M.. Interaction of 31 β-lactam antibiotics with the H +/peptide symporter PEPT2: Analysis of affinity constants and comparison with PEPT1. Eur. J. Pharm. Biopharm. 59 (2005) 17–24. doi: https://doi.org/10.1016/j.ejpb.2004.07.008. 10.1016/j.ejpb.2004.07.008 15567297

[21] Mariappan T.T. Singh S.. Evidence of efflux-mediated and saturable absorption of rifampicin in rat intestine using the ligated loop and everted gut sac techniques. Mol. Pharm. 1 (2004) 363–367. doi: https://doi.org/10.1021/mp049937n. 10.1021/mp049937n 16026006

[22] Bermejo M. Hens B. Dickens J. Mudie D. Paixão P. Tsume Y. Shedden K. Amidon G.L.. A mechanistic physiologically-based biopharmaceutics modeling (PBBM) approach to assess the in vivo performance of an orally administered drug product: From IVIVC to IVIVP. Pharmaceutics 12 (2020). doi: https://doi.org/10.3390/pharmaceutics12010074. 10.3390/pharmaceutics12010074 PMC7023481 31963448

[23] Jereb R. Opara J. Legen I. Petek B. Grabnar-Peklar D.. In vitro–In vivo Relationship and Bioequivalence Prediction for Modified-Release Capsules Based on a PBPK Absorption Model. AAPS PharmSciTech 21 (2020). doi: https://doi.org/10.1208/s12249-019-1566-x. 10.1208/s12249-019-1566-x 31820131

[24] de Miranda P. Blum M.. Pharmacokinetics of acyclovir after intravenous and oral administration. J. Antimicrob. Chemother. 12 (1983) 29–37. doi: https://doi.org/10.1093/jac/12.suppl_b.29. 10.1093/jac/12.suppl_b.29 6355048

[25] Foulds G. Shepard R. Johnson R.. The pharmacokinetics of azithromycin in human serum and tissues. - PubMed - NCBI. J. Antimicrob. Chemother. 25 (1990) 73–82. doi: https://doi.org/10.1093/jac/25.suppl_a.73. 10.1093/jac/25.suppl_a.73 2154441

[26] Faulkner R.D. Fernandez P. Lawrence G. Sia L.L. Falkowski A.J. Weiss A.I. Yacobi A. Silber B.M.. Absolute bioavailability of cefixime in man. J. Clin. Pharmacol. 28 (1988) 700–706. doi: https://doi.org/10.1002/j.1552-4604.1988.tb03203.x. 10.1002/j.1552-4604.1988.tb03203.x 3216036

[27] Hull J.H. Findlay J.W.A. Rogers J.F. Welch R.M. Butz R.F. Bustrack J.A.. An evaluation of the effects of smoking on codeine pharmacokinetics and bioavailability in normal human volunteers. Drug Intell. Clin. Pharm. 16 (1982) 849–854. doi: https://doi.org/10.1177/106002808201601107. 10.1177/106002808201601107 7173046

[28] Gandhi Y. Eley T. Fura A. Li W. Bertz R.J. Garimella T.. Daclatasvir: A Review of Preclinical and Clinical Pharmacokinetics. Clin. Pharmacokinet. 57 (2018) 911–928. doi: https://doi.org/10.1007/s40262-017-0624-3. 10.1007/s40262-017-0624-3 29353349

[29] Wolfe C. Hicks C.. Profile of darunavir in the management of treatment-experienced HIV patients. HIV/AIDS - Res. Palliat. Care. (2009) 13. doi: https://doi.org/10.2147/hiv.s4842. 10.2147/hiv.s4842 PMC3218680 22096376

[30] Dolutegravir FDA. Decisional Review for NDA 204790., 2013. https://www.accessdata.fda.gov/scripts/cder/daf/index.cfm?event=overview.process&varApplNo=204790 (accessed May 6, 2020).

[31] FDA, Sustiva approval history: Application number 20-360., 2011. https://www.accessdata.fda.gov/drugsatfda_docs/nda/98/20972biopharm_review.pdf.

[32] Nyberg H.B. Draper H.R. Garcia-Prats A.J. Thee S. Bekker A. Zar H.J. Hooker A.C. Simon Schaaf H. McIlleron H. Hesseling A.C. Denti P., Population pharmacokinetics and dosing of ethionamide in children with tuberculosis. Antimicrob. Agents Chemother. 64 (2020). doi: https://doi.org/10.1128/AAC.01984-19. 10.1128/AAC.01984-19 PMC7038277 31871093

[33] Smith D. Lin E. Benet L.. Absorption and disposition of furosemide in healthy volunteers, measured with a metabolite-specific assay. Drug Metab. Dispos. 8 (1980) 337–342. https://www.ncbi.nlm.nih.gov/pubmed/?term=6107232 (accessed May 6, 2020). 6107232

[34] Mihaly G. Ward S. Edwards G. Nicholl D. Orme M. Breckenridge A.. Pharmacokinetics of primaquine in man. I. Studies of the absolute bioavailability and effects of dose size. Br. J. Clin. Pharmacol. 19 (1985) 745–750. doi: https://doi.org/10.1111/j.1365-2125.1985.tb02709.x. 10.1111/j.1365-2125.1985.tb02709.x 4027117 PMC1463857

[35] Almond D.S. Szwandt I.S.F. Edwards G. Lee M.G. Winstanley P.A.. Disposition of intravenous pyrimethamine in healthy volunteers. Antimicrob. Agents Chemother. 44 (2000) 1691–1693. doi: https://doi.org/10.1128/AAC.44.6.1691-1693.2000. 10.1128/AAC.44.6.1691-1693.2000 10817730 PMC89934

[36] Brainard D.M. Wenning L.A. Stone J.A. Wagner J.A. Iwamoto M.. Clinical pharmacology profile of raltegravir, an HIV-1 integrase strand transfer inhibitor. J. Clin. Pharmacol. 51 (2011) 1376–402. doi: https://doi.org/10.1177/0091270010387428. 10.1177/0091270010387428 21209233

[37] Loos U. Musch E. Jensen J.C. Mikus G. Schwabe H.K. Eichelbaum M.. Pharmacokinetics of oral and intravenous rifampicin during chronic administration. Klin. Wochenschr. 63 (1985) 1205–1211. doi: https://doi.org/10.1007/BF01733779. 10.1007/BF01733779 4087830

[38] Barditch-Crovo P. Deeks S.G. Collier A. Safrin S. Coakley D.F. Miller M. Kearney B.P. Coleman R.L. Lamy P.D. Kahn J.O. McGowan I. Lietman P.S.. Phase I/II trial of the pharmacokinetics, safety, and antiretroviral activity of tenofovir disoproxil fumarate in human immunodeficiency virus-infected adults. Antimicrob. Agents Chemother. 45 (2001) 2733–2739. doi: https://doi.org/10.1128/AAC.45.10.2733-2739.2001. 10.1128/AAC.45.10.2733-2739.2001 11557462 PMC90724

[39] Bransford P. Cook J. Gupta M. Haertter S. He H. Ju R. Kanodia J. Lennernäs H. Lindley D. Polli J.E. Wenning L. Wu Y.. ICH M9 Guideline in Development on Biopharmaceutics Classification System-Based Biowaivers: An Industrial Perspective from the IQ Consortium. Mol. Pharm. 17 (2020) 361–372. doi: https://doi.org/ 10.1021/acs.molpharmaceut.9b01062. 10.1021/acs.molpharmaceut.9b01062 31846335

[40] Sjögren E. Thörn H. Tannergren C.. In Silico Modeling of Gastrointestinal Drug Absorption: Predictive Performance of Three Physiologically Based Absorption Models. Mol. Pharm. 13 (2016) 1763–1778. doi: https://doi.org/10.1021/acs.molpharmaceut.5b00861. 10.1021/acs.molpharmaceut.5b00861 26926043

