# Supplementary material for: Global testing of a consensus solubility assessment to enhance robustness of the WHO biopharmaceutical classification system
Source: ADMET DMPK. 2020 Oct 7;9(1):23–39. doi: 10.5599/admet.850 (PMC8923308; doi:10.5599/admet.850)
Supplement: Supplementary file 1 [file admet-9-850-S1.pdf]

Supporting Information for

Global testing of a consensus solubility assessment to enhance robustness of the WHO biopharmaceutical classification system

ADMET and DMPK (2020) doi: https://dx.doi.org/10.5599/admet.850

Valeria Gigante[1,*], Giovanni M. Pauletti[2], Sabine Kopp[1], Minghze Xu[3], Isabel Gonzalez-Alvarez[10], Virginia Merino[4], Michelle P. McIntosh[5], Anita Wessels[6], Beom-Jin Lee[7], Kênnia Rocha Rezende[8], Gerhard K.E. Scriba[9], Gaurav P. S.Jadaun[11], Marival Bermejo[10]

[1]*Norms and Standards for Pharmaceuticals, World Health Organization, Geneva, Switzerland;*
[2]*Department of Pharmaceutical and Administrative Sciences, St. Louis College of Pharmacy, St. Louis, Missouri, USA*
[3]*Institute for Chemical Drug Control, China National Institutes for Food and Drug Control, Beijing, China*
[4]*Department of Pharmaceutics and Pharmaceutical Technology and Parasitology, University of Valencia, Valencia,*
Spain

[5]*Drug Delivery Disposition and Dynamics, Monash Institute of Pharmaceutical Sciences, Monash University, Parkville, Australia*
[6]*North_West University, School of Pharmacy, Potchefstroom, South Africa*
[7]*College of Pharmacy and Institute of Pharmaceutical Science and Technology, Ajou University, Suwon, Republic of Korea*
[8]*Faculty of Pharmacy, Federal University of Goiás, Brazil*
[9]*Department of Pharmaceutical Chemistry, Friedrich Schiller-University, Jena, Germany*
[11]*Indian Pharmacopoeia Commission, Ministry of Health & Family Welfare, Govt. of India, Ghaziabad, India*
[10]*Department of Engineering: Pharmacy section, Universidad Miguel Hernández de Elche, Alicante, Spain*

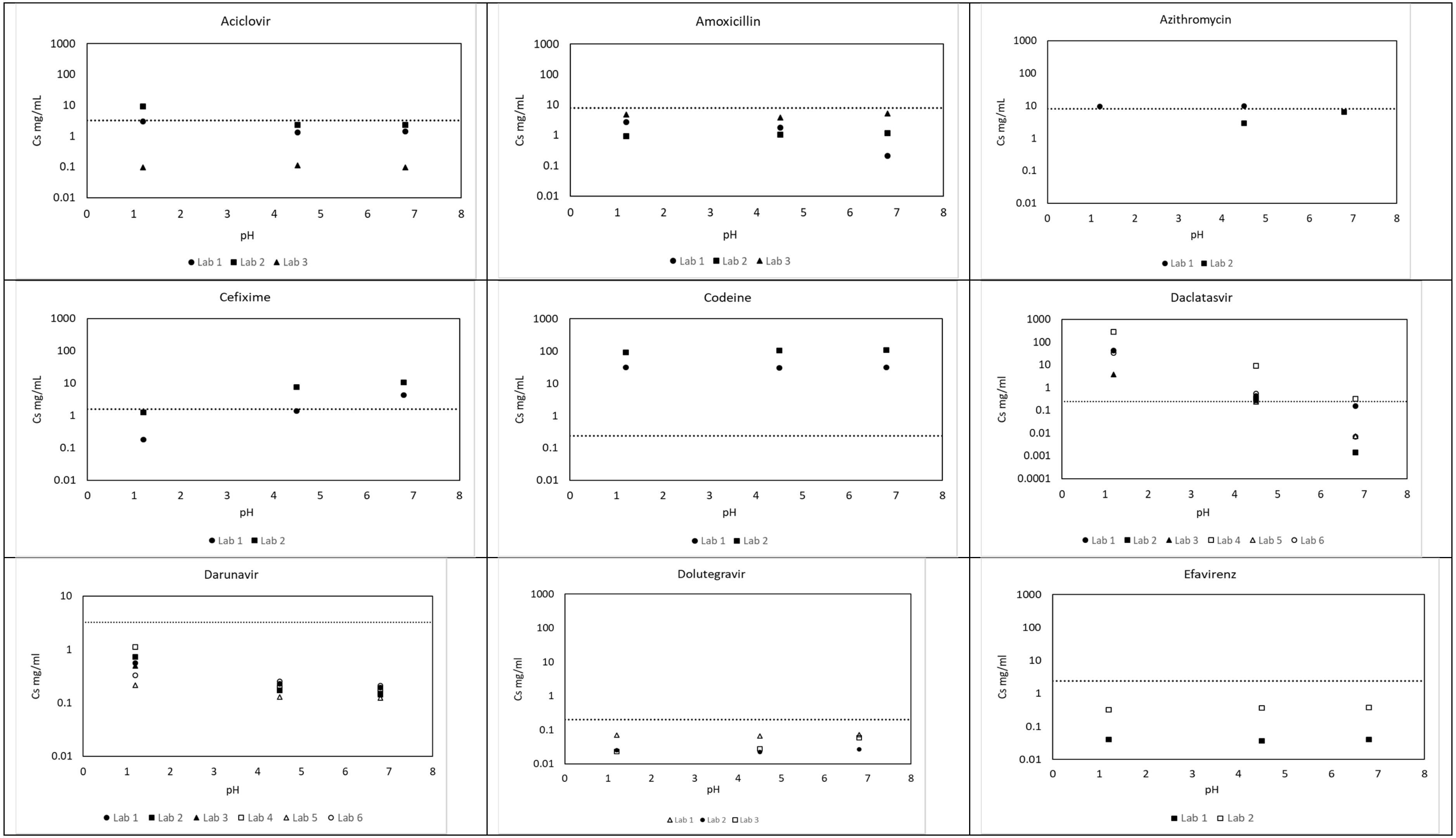
Aciclovir
Cs mg/mL
1000
100
10
1
0.1
0.01
0 1 2 3 4 5 6 7 8
pH
Lab 1 Lab 2 Lab 3
Amoxicillin
Cs mg/mL
1000
100
10
1
0.1
0.01
0 1 2 3 4 5 6 7 8
pH
Lab 1 Lab 2 Lab 3
Azithromycin
Cs mg/mL
1000
100
10
1
0.1
0.01
0 1 2 3 4 5 6 7 8
pH
Lab 1 Lab 2
Cefixime
Cs mg/mL
1000
100
10
1
0.1
0.01
0 1 2 3 4 5 6 7 8
pH
Lab 1 Lab 2
Codeine
Cs mg/mL
1000
100
10
1
0.1
0.01
0 1 2 3 4 5 6 7 8
pH
Lab 1 Lab 2
Daclatasvir
Cs mg/ml
1000
100
10
1
0.1
0.01
0.001
0.0001
0 1 2 3 4 5 6 7 8
pH
Lab 1 Lab 2 Lab 3 Lab 4 Lab 5 Lab 6
Darunavir
Cs mg/ml
10
1
0.1
0.01
0 1 2 3 4 5 6 7 8
pH
Lab 1 Lab 2 Lab 3 Lab 4 Lab 5 Lab 6
Dolutegravir
Cs mg/ml
1000
100
10
1
0.1
0.01
0 1 2 3 4 5 6 7 8
pH
Lab 1 Lab 2 Lab 3
Efavirenz
Cs mg/ml
1000
100
10
1
0.1
0.01
0 1 2 3 4 5 6 7 8
pH
Lab 1 Lab 2

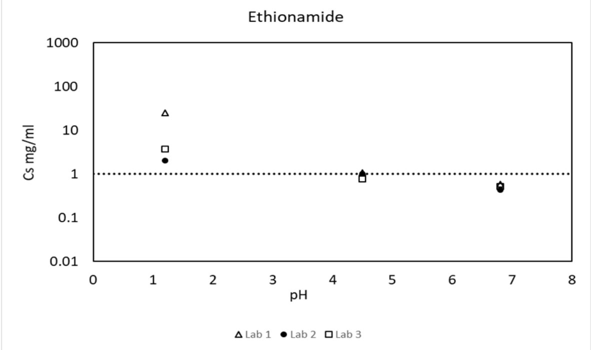
Ethionamide
1000
100
10
1
0.1
0.01
Cs mg/ml
0 1 2 3 4 5 6 7 8
pH
Lab 1 Lab 2 Lab 3

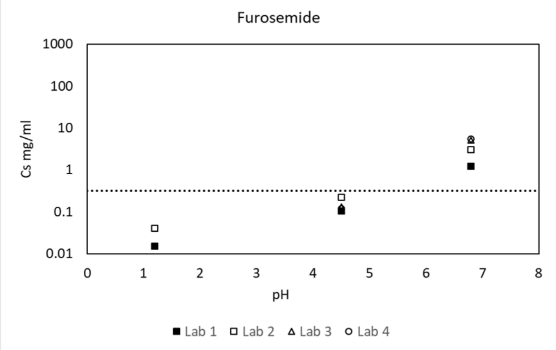
Furosemide
1000
100
10
1
0.1
0.01
Cs mg/ml
0 1 2 3 4 5 6 7 8
pH
Lab 1 Lab 2 Lab 3 Lab 4

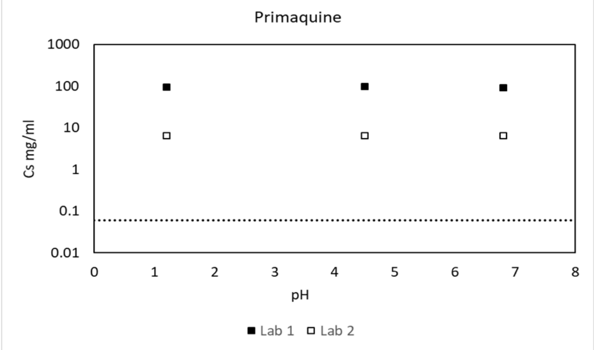
Primaquine
1000
100
10
1
0.1
0.01
Cs mg/ml
0 1 2 3 4 5 6 7 8
pH
Lab 1 Lab 2

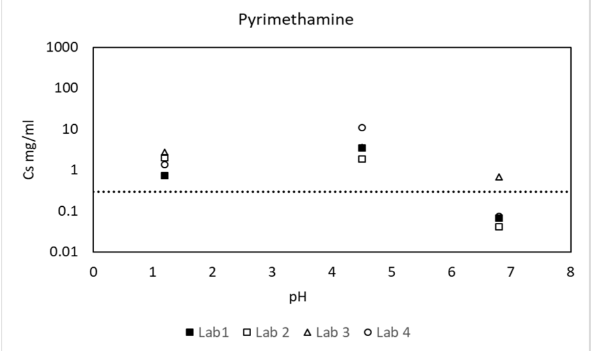
Pyrimethamine
1000
100
10
1
0.1
0.01
Cs mg/ml
0 1 2 3 4 5 6 7 8
pH
Lab1 Lab 2 Lab 3 Lab 4

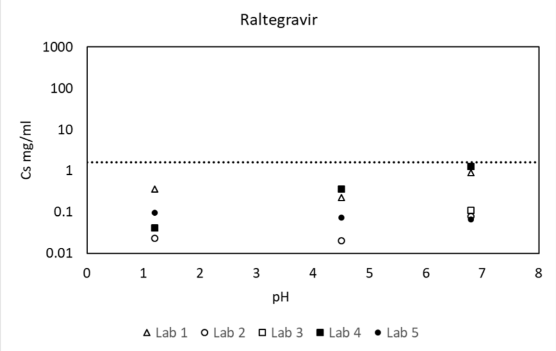
Raltegravir
1000
100
10
1
0.1
0.01
Cs mg/ml
0 1 2 3 4 5 6 7 8
pH
Lab 1 Lab 2 Lab 3 Lab 4 Lab 5

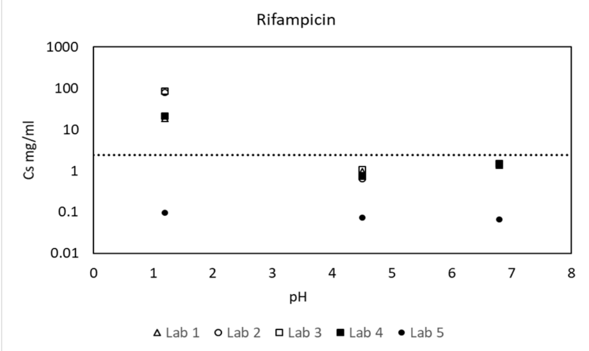
Rifampicin
1000
100
10
1
0.1
0.01
Cs mg/ml
0 1 2 3 4 5 6 7 8
pH
Lab 1 Lab 2 Lab 3 Lab 4 Lab 5

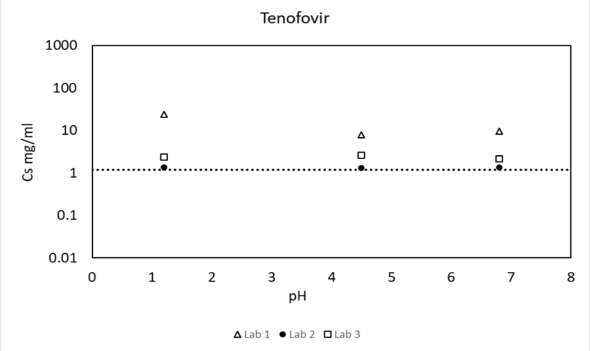
Tenofovir
1000
100
10
1
0.1
0.01
Cs mg/ml
0 1 2 3 4 5 6 7 8
pH
Lab 1 Lab 2 Lab 3

		ADMET Predictor© 9.5			GastroPlus© 9.7													Clinical Data			
		Phys.-Chem. Properties		Metabolism	Predicted Solubility			Permeability [cm/s *10e4]	Pharmacokinetic Simulations									Pharmacokinetic Results			
API	Salt Form	pKa	logP	Predicted Affinity	pH 1.2	pH 4.5	pH 6.8	Peff human	Highest Dose [mg]	Simulation time [hrs]	Predicted fa%	Predicted F%	Permeability Classification	Highest Strength [mg]	Simulation time [hrs]	Predicted fa%	Predicted F%	Dose [mg]	F%	Ref.	Permeability Classification
Aciclovir	N/A	9.58 (acid); 2.42 (base); 0.42 (base); -1.02 (base)	-1.40	CYP1A2	3.60	1.78	1.77	0.45	800	24	53.5	51.9	LP	200	24	54.3	52.2	200	20.0	#22	LP
Amoxicillin	trihydrate	9.88 (acid); 7.17 (base); 3.12 (acid)	-1.94	N/A	119.44	3.39	4.64	0.27	3000	24	40.4	39.7	LP	500	24	43.9	42.7	3000	45.0	#19	LP
Azithromycin	dihydrate	8.72 (base; 7.63 (base)	3.39	CYP3A4	135.21	135.31	155.45	0.27	2000	24	62.6	61.0	LP	500	48	62.6	61.0	500	37.0	#23	LP
Cefixime	trihydrate	10.16 (acid); 4.11 (base); 3.21 (acid); 2.52 (acid); -0.96 (base); -4.02 (base)	-1.23	N/A	16.48	51.10	73.10	0.38	400	24	54.5	53.3	LP	400	24	54.5	53.3	400	40.2	#24	LP
Codeine	phosphate hemihydrate	8.63 (base)	1.61	CYP1A2, 2C9, 2D6, 3A4	91.59	91.60	92.94	3.26	60	24	99.0	98.9	HP	30	24	99.0	98.9	60	54.8	#25	LP
Daclatasvir	dihydrochloride	11.33 (acid); 10.9 (acid); 10.54 (acid); 10.1 (acid); 4.28 (base); 3.64 (base)	4.06	CYP3A4	17.61	0.02	0.01	0.64	60	24	97.0	97.0	HP	60	24	97.0	97.0	60	67.0	#26	LP
Darunavir	ethanolate	11.32 (acid); 3.37 (base)	1.93	CYP3A4	14.56	0.11	0.10	0.37	800	24	23.4	23.3	LP	800	24	23.4	23.3	600	37.0	#27	LP
Dolutegravir	N/A	10.5 (acid); 4.58 (acid)	1.20	N/A	0.39	0.72	53.53	1.96	50	24	96.2	95.8	HP	50	24	96.2	95.8	50	31.0	#28	LP
Efavirenz	N/A	9.66 (acid)	4.19	CYP3A4	0.00	0.00	0.00	5.30	600	24	5.8	5.8	LP	600	24	5.8	5.8	400	19.5	#29	LP
Ethionamide	N/A	11.09 (acid); 4.25 (base); 0.00881 (base)	1.26	CYP1A2, 2C9, 2C19, 2D6, 3A4	48.94	1.59	1.02	3.51	1000	24	99.0	98.6	HP	250	24	98.9	98.5	250	83.3	#30	LP
Furosemide	N/A	9.97 (acid); 3.97 (acid); -0.23 (base)	2.07	CYP2C9	0.07	0.28	21.26	0.53	80	24	75.9	74.7	LP	40	24	75.9	74.8	80	42.8	#31	LP
Primaquine	phosphate	9.92 (base); 3.88 (base); 0.46 (base)	2.86	CYP 1A2, 2D6	100.43	105.16	84.98	1.89	15	24	97.7	97.6	HP	15	24	97.7	97.6	15	96.0	#32	HP
Pyrimethamine	N/A	6.57 (base); 0.99 (base); -2.54 (base); -3.35 (base)	2.53	CYP1A2, 2C9, 2C19, 2D6	23.31	4.27	0.06	2.11	75	24	99.9	99.8	HP	25	24	99.9	99.8	20	50.0	#33	LP
Raltegravir	potassium	11 (acid); 10.26 (acid); 5.12 (acid); 1.53 (base); -1.28 (base); -3.12 (base)	1.97	CYP2C9	1.06	0.42	16.40	1.86	400	24	97.5	97.1	HP	400	24	97.5	97.1	400	30.0	#34	LP
Rifampicin	N/A	11.86 (acid); 11.02 (acid); 10.08 (acid); 7.84 (acid); 6.91 (base); 2.39 (base); -1.54 (base)	2.53	CYP3A4	47.01	26.22	0.24	0.23	750	24	37.9	37.7	LP	300	24	65.6	65.0	600	93.0	#35	HP
Tenofovir disoproxil	fumarate	6.23 (acid); 4.48 (base); 1.52 (acid); 0.62 (base); -2.2 (base); -3.18 (base)	-1.34	N/A	34.50	19.60	203.75	0.42	300	24	57.2	55.6	LP	300	24	57.2	55.6	300	25.0	#36	LP
